# Automated Diagnostics: Advances in the Diagnosis of Intestinal Parasitic Infections in Humans and Animals

**DOI:** 10.3389/fvets.2021.715406

**Published:** 2021-11-23

**Authors:** Sandra Valéria Inácio, Jancarlo Ferreira Gomes, Alexandre Xavier Falcão, Bianca Martins dos Santos, Felipe Augusto Soares, Saulo Hudson Nery Loiola, Stefani Laryssa Rosa, Celso Tetsuo Nagase Suzuki, Katia Denise Saraiva Bresciani

**Affiliations:** ^1^São Paulo State University (Unesp), School of Veterinary Medicine, Araçatuba, Brazil; ^2^School of Medical Sciences, University of Campinas (UNICAMP), Campinas, Brazil; ^3^Institute of Computing (IC), University of Campinas (UNICAMP), Campinas, Brazil

**Keywords:** automated, parasite, protozoan, animal, human, gastrointestinal, technological progress, helminths

## Abstract

The increasingly close proximity between people and animals is of great concern for public health, given the risk of exposure to infectious diseases transmitted through animals, which are carriers of more than 60 zoonotic agents. These diseases, which are included in the list of Neglected Tropical Diseases, cause losses in countries with tropical and subtropical climates, and in regions with temperate climates. Indeed, they affect more than a billion people around the world, a large proportion of which are infected by one or more parasitic helminths, causing annual losses of billions of dollars. Several studies are being conducted in search for differentiated, more sensitive diagnostics with fewer errors. These studies, which involve the automated examination of intestinal parasites, still face challenges that must be overcome in order to ensure the proper identification of parasites. This includes a protocol that allows for elimination of most of the debris in samples, satisfactory staining of parasite structures, and a robust image database. Our objective here is therefore to offer a critical description of the techniques currently in use for the automated diagnosis of intestinal parasites in fecal samples, as well as advances in these techniques.

## Introduction

Parasitic infectious diseases pose an important public health problem, particularly in developing countries, where basic sanitation services are often poor, and diseases are aggravated by environmental factors such as temperature, type of soil, seasonal precipitation and overall climate in each geographic region (1).

The increasingly close proximity between people and their pet animals, which are kept for companionship, entertainment and emotional support, also increases the risk of exposure to infectious diseases, since animals are carriers of more than 60 zoonotic agents (2). Despite advances in tools for the management and control of parasitic diseases, veterinarians and other health professionals still consider the occurrence of intestinal parasites in pet animals very important (3–6).

These diseases are included in the list of “Neglected Tropical Diseases,” causing losses in countries with tropical and subtropical climates and in regions with temperate climates and affecting more than a billion people, one sixth of the world population, a large proportion of which are infected by one or more helminths, causing billions of dollars in losses every year (7).

The agents responsible for amebiasis, ascariasis, hookworms and trichuriasis are among the ten most prevalent infectious parasites in the human population worldwide. However, although their mortality rate is low, complications are common and many cases require hospitalization (8).

Malabsorption, diarrhea, hemorrhage, impaired work capacity, reduced growth rate and impaired cognitive skills are serious health and social problems linked to intestinal parasitic infections that cause serious economic burdens on populations (8).

Gastrointestinal parasites are common in dogs and cats and can cause major damage to the gastrointestinal tract, although some animals may be asymptomatic (9–13). The most common endoparasites of dogs and cats, which can be a source of transmission to humans and are considered zoonotic and of concern for public health (10), are *Giardia* spp., *Toxocara* spp., and *Ancylostoma* spp. (4, 9, 10).

Giardiasis can be asymptomatic, but it can also cause acute or chronic diarrhea, in addition to delayed growth in humans and animals, as well as decreased cognitive functions and chronic fatigue. It can also lead to post-infectious functional gastrointestinal disorders, such as irritable bowel syndrome and functional dyspepsia (14).

Parasites of the genus *Toxocara* cause infections that are often asymptomatic, but when their larvae migrate from the small intestine into the bloodstream, they reach the tissues and cause the syndrome called Visceral Larva Migrans (VLM), which can migrate to the eyes and result in Ocular Larva Migrans (OLM) syndrome. Other pathologies associated with this parasite are neurotoxocariasis and covert toxocariasis (15, 16). Commonly affected organs are liver, lungs, heart, brain and eyes, causing an intense inflammatory response, eosinophilia, and high levels of total IgE (15, 17–20).

Furthermore, parasites of the species *Ancylostoma braziliense* and *Ancylostoma caninum* can cause Cutaneous Larva Migrans (21), with infective larvae penetrating the skin and moving to the dermis, causing inflammation with severe pruritus (13, 22, 23).

Helminth larvae in dogs are present in the intestine, where they produce thousands of eggs that are excreted in feces and contaminate the environment. Transmission occurs through contaminated water, ingestion of poorly washed or cooked greens and vegetables, and through ingestion by children who play on contaminated soil and touch their mouths with dirty hands. Thus, helminth eggs, cysts and oocysts of protozoa are excreted in the feces of infected animals, contaminating the environment, which is the main source of infections in animals and humans (8, 24).

To detect the presence of parasites in the stool, it is necessary to make use of parasitological laboratory techniques. The techniques most frequently used are Flotation in Saturated Sodium Chloride Solution (25), Centrifugal Flotation in Saturated Zinc Sulfate Solution (26) and Spontaneous Sedimentation (27). These techniques are used mainly due to their low cost and because they are practical and direct (13, 28–32).

Notably, the literature reports that the diagnostic sensitivity of the above-mentioned analytical techniques may be low to moderate. This limitation may be attributed to differences these in techniques, from sample collection to laboratory processing. The interpretation of laboratory analyses may be impaired if performed by a professional with little experience in identifying the wide variety of existing parasites (30, 33–35). These challenges must be overcome so that a more precise technique with specific results, involving a wide variety of parasites, can be developed (36).

Such good results can be achieved by using a new technique known as the TF-Test (Three Fecal Test), which has performed well, showing good sensitivity in studies with fecal samples from humans, cattle, sheep and dogs. This can be accomplished by means of triple sampling, suitable preservatives in sample collection tubes, transport, homogenization and an appropriate protocol (29, 30, 33–35, 37–39).

**Figure 1:**
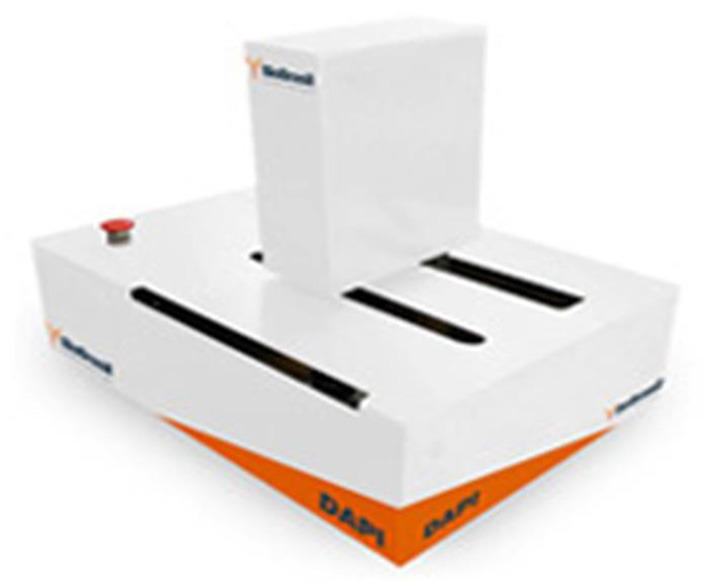
Image courtesy of Laboratory of Image Data Science (LIDS)- Unicamp. Techniques commonly used in routine laboratory procedures and the tendency toward automated diagnostics.

An extensive scientific and technological study for automated diagnostics is under development, aiming to reduce the types of errors described above. The system consists of a parasitology protocol, personal computer, and a microscope coupled to a high resolution digital camera equipped with an appropriate optical tube and platinum motorized dome (40, 41). This new system is called “Automated Diagnosis of Intestinal Parasites” [DAPI] (Figure 1) (37, 40, 41).

In the field of veterinary medicine, this new protocol has shown good performance in the diagnosis of intestinal parasites in dogs. This justifies the continuing development of automated diagnostics, which requires a protocol to obtain a cleaner slides, free of impurities and debris, enabling the computer system to more accurately identify parasite structures (38).

Immunological and molecular techniques, which are widely used in epidemiological surveys, scientific research and for the description of parasite species, are expensive, thus restricting their use in laboratory routines (33, 35).

**Figure 2:**
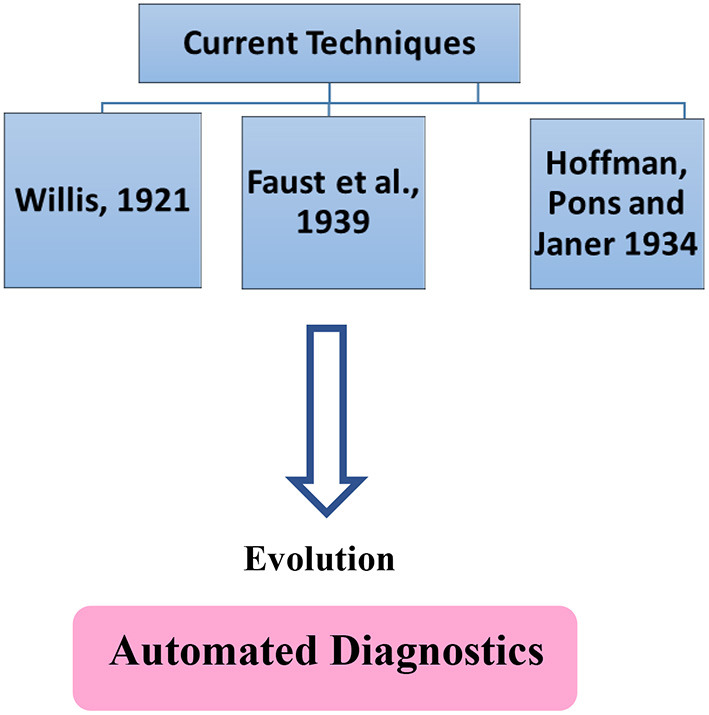
Techniques commonly used in routine laboratory procedures and the tendency towards automated diagnostics.

Therefore, we emphasize the importance of an initial protocol, i.e., one that enables parasite structures to be more clearly visible in order to assist the automatic identification system. Traditionally accepted techniques widely published in the literature leave behind a lot of debris and impurities that end up hindering this identification. Hence, a technique that includes an initial protocol aimed at reducing these problems is ideal for this identification through good software programs. Our objective is therefore to critically describe the techniques currently in use for the automated diagnosis of intestinal parasites in fecal samples, as well as advances in these techniques, thus demonstrating the necessary requirements for their automation (Figure 2).

## Article Retrieval Method

The articles were retrieved from the SciELO, ScienceDirect, and Google Scholar databases, and were selected after reading their contents.

## Development

### Advances and Limits of Computational Diagnostic Techniques for the Identification of Intestinal Parasites

Several studies are being conducted to devise differentiated, more sensitive diagnostics with fewer errors. These studies, which involve the automated examination of intestinal parasites (Table 1), still face challenges that must be overcome in order to ensure the proper identification of parasites. This includes a protocol for the preparation of microscope slides containing less debris, good staining of parasite structures, as well as a robust image database (38).

**Table 1:** Articles pertaining to the automatic identification of gastrointestinal parasites according to the computational techniques employed.

**Preparation Protocol**	**No. of Parasites**	**Software**	**Image input devices**	**% Accuracy**	**% Sensitivity**	**Authors/year**
Centrifugation-sedimentation	4	SCILAB Computational platform	Optical microscope (Dialux Leitz Weitz) and camera (Olympus C-3030)	–	99–100%	(42)
Not described	16	MATLAB (MathWorks, Natick, MA)	Kansas State University Database	NA	NA	(41)
Not described	1	Gimp Adobe Photoshop	Optical microscope (Nikon Eclipse E800) and camera (Nikon Coolpix 4500)	85.75 %	–	(43)
Saturated flotation fluid	7	Leica Quantimet 500 MC	Not described	93%	–	(44)
Not described	15	MATLAB (MathWorks, Natick, MA)	Not described	93.49%	–	(45)
Not described	2	AnalySIS (Olympus Soft Imaging Solutions)	Olympus BX41TF-FL_CCD Microscope, Olympus XC50 camera	93–94%	–	(46)
Not described	2	Diagnosis Support System (DSS)	Nikon Eclipse E200 microscope	81.86%	–	(47)
Ethyl acetate technique	4	MATLAB (MathWorks, Natick, MA)	MoMic digital microscope scanner and camera module (CM6787-O500BA-E, TRULY Optoelectronics)	–	83.3–100%	(48)
TF-GII/Dog technique	4	Automated Diagnosis of Intestinal Parasites System (DAPI)	Automated Diagnosis of Intestinal Parasites (DAPI)	–	80.88%	(38)
Ethyl acetate sedimentation (SED-CONNECT concentration kits)	13	SediMAX System	Cuvette-based automated microscopy analyzer (sediMAX 1)	–	91.66–100%	(49)
Ethyl acetate sedimentation (SED-CONNECT concentration kits)	7	sediMAX System	Cuvette-based automated microscopy analyzer (sediMAX 2)	–	100%	(50)
Not described	7	MATLAB (MathWorks, Natick, MA)	Carl Zeiss AxioLab A1 optical microscope and Imaging Development Systems UI-1480LE USB2 color camera	–	80–90%	(51)
McMaster flotation technique	6	MATLAB (MathWorks, Natick, MA)	Portable automated microscope	92–96%	72–100%	(52)
Saturated flotation technique—Mini Parasep® and Ovassay® (spontaneous and centrifuge)	4	VETSCAN IMAGYST	MoticEasyScan One® digital slide scanner (Motic, Kowloon Bay, Hong Kong)	93–94%	75.8–100%	(53)
Ethyl acetate sedimentation-centrifugation technique (TF-Test®)	15	Automated Diagnosis of Intestinal Parasites (DAPI) system	Automated Diagnosis of Intestinal Parasites (DAPI)	87.8–94%	–	(54)
Not described	15	Not described	Customized motorized microscope with digital camera to capture images from slides	82–98%	–	(55)
Not described	15	Not described	Not described	99–99%	40–99%	(56)
Not described	15	Not described	Public and private clinical laboratory (databases available online)	100%	–	(57)
Immunofluorescence assay (IFA)	2	BrainMaker Professional (California Scientific Software)	Microscopes: Olympus BH-2, Zeiss Axioplan 2, or Olympus BX-50	91–99%	–	(58)
Not described	7	MATLAB (MathWorks, Natick, MA)	Olympus microscope, JVC Video camera	86–90%	–	(59)
Not described	9	Helminth Egg Automatic Detector (HEAD)	Microscope: Carl Zeiss AxioLab A1; Imaging Development Systems UI-1480LE-C camera	96%		(51)
Standard trichrome staining	8	Pannoramic Viewer software (3DHISTECH Ltd.)	Pannoramic 250 Flash III (3DHISTECH, Budapest, Hungary)	98–98%	–	(60)
Salt-sugar flotation and Wisconsin sugar flotation technique	1	Image analysis package Ks400 (Carl Zeiss Vision, Germany)	Coolpix 950 digital camera (Nikon) equipped with a Nikon binocular eyepiece tube (SMZ800)	–	–	(61)
Modified McMaster, and Mini-Flotac techniques	1	ImageJ software (National Institute of Health, Bethesda, MD, USA)	Smartphone prototype	32%	94%	(62)
Sugar flotation technique (saturated solution of sodium chloride with glucose)	5	GIPS® TAR 4.11 (Image House, Copenhagen, Denmark) (Vision Research, Copenhagen, Denmark)	Leitz Laborlux D microscope	81%	–	(63)
Flotation in saturated sodium chloride solution	1	ImageJ software (National Institute of Health, Bethesda, MD, USA)	Nokia Lumia 1020 mobile phone (Microsoft Corp., Auckland, New Zealand)	NA	NA	(64)
Commercial fluorescent monoclonal antibody staining kit (*Cryptosporidium*/*Giardia* IF Test; TechLab, Blacksburg, Va.)	1	Neural Network Approach program (BrainMaker Professional; California Scientific Software, Nevada City, Calif.)	BH-2 Olympus microscope; color digital camera (SPOT CCD; SP100; Diagnostic Instruments, Inc., Sterling Heights, Mich., USA)	81–97%	–	(65)

*NA, Not available*.

Over the last decade, researchers have been working on digital image processing models and pattern techniques for the automatic recognition of parasite eggs in microscopic images. The main objective is to reduce human errors that occur in the diagnosis of fecal parasites, and to produce faster and highly accurate results. These research efforts clearly illustrate the crucial importance of the automated identification of intestinal parasites in producing efficient and reliable results (43).

Intestinal parasites are considered of great interest in the implementation of algorithms for automated identification based on diagnostic imaging, because these organisms have stages of development with well-defined and reasonably homogeneous morphology (44).

Notably, the use of automated diagnostic techniques to identify and count eggs, cysts, and oocysts of helminths and protozoa is also very important. Moreover, with regard to the volume of samples processed in a short time, automated diagnostics offer high precision in the identification of host positivity and parasite load, allied to less fatigue and less time spent counting eggs on a computer screen when compared to the traditional microscope process (45).

Given the increasing use of computational technologies, the production and storage of visual and textual databases is essential. This means that an effective and efficient tool is needed to obtain such information to satisfy automation requirements (46, 47). Thus, image annotation methods have been based on several types of supervised classifiers (48), Bayesian classifier (49–51), Support Vector Machines (SVM) (52, 53), Artificial Neural Networks (ANNs) (66, 67), k-nearest neighbor (k-NN) (68, 69), Decision Tree (DT) (51, 52, 60), and Optimum-Path Forest (OPF) (61, 62).

ANNs implement algorithms to reproduce the processing functions of neural networks, in which neurons are arranged in layers (each neuron being connected to all the other neurons in the preceding layer) and process the information. The information is applied to an activation function and passes on signals to other neurons within the system. Using this structure of interconnected neurons, neural networks undergo a training procedure in which they “learn” how to discern patterns in data (63, 64). Backpropagation, a common learning algorithm employed by ANN, involves two steps. The first step is direct processing of input data through the neurons, which produce a predicted solution, while the second step involves the correction of weights within the layers of neurons to minimize the errors of the predicted solution relative to the true solution (63).

Initial studies on automated diagnostics involved the analysis of fecal samples from cattle (56, 65, 70) and pigs (71, 72). These studies of fecal samples from livestock differentiated the parasite eggs based on their characteristics (56, 65, 70–72). The use of the parameter of texture, which is the variation of the gray level in digital image processing, has also been investigated, and improves the classification result when used together with the characteristics of shape and size (70).

In a preliminary study to detect helminths using digital image processing techniques (73) and an artificial neural network (ANN) system (74), 82 images of seven human parasites were acquired. This study achieved an execution and identification rate of 84% and 83% respectively, and proved the applicability of the developed algorithms to the fully automatic examination system (75). This same ANN system was used in immunofluorescence staining (76) and 4′,6-diamidino-2-phenylindole [DAPI] (77) to identify the protozoan *Cryptosporidium* spp. in order to solve errors in mechanical identification resulting from factors such as technician fatigue and inexperience (77, 78).

The study generated from automated diagnostics has also evolved to the counting of bovine parasitic nematode eggs. The work in question involved a comparison of two techniques, Salt-Sugar Flotation (SSF) and Wisconsin Sugar Flotation (WSF). The former technique proved to be significantly better than the latter, since it allows a larger number of samples to be processed and provides a high degree of precision. Although these techniques use solutions that are close to saturation, they were diluted in the research in question (45).

Another study involved working on images of the protozoa *Cryptosporidium* spp. and *Giardia* spp. taken from slides stained with fluorescein-labeled monoclonal antibodies. The images were taken with a color digital camera, and the color information was discarded through dither filtering. These two protozoans were detected using Artificial Neural Networks (ANN), which correctly identified 91.8 of the images of the *Cryptosporidium* oocyst and 99.6% the *Giardia* cyst, respectively, indicating that it can be extremely useful in automated diagnostics (66). The above-mentioned techniques have several limitations, such as the difficulty of quantifying morphological characteristics, allied to the high complexity of the algorithms (44).

The purpose of image analysis is to classify and recognize objects of interest in digital images. This can be done in several ways, e.g., by identifying the colors, textures, shapes, movements and position of the objects in the images (44). Thus, different species of *Eimeria* found in farmyard chickens were included in the study of automated identification. This involved working with three groups of features, namely, characterization of the curvature, size, and symmetry of the internal structure, and quantification, i.e., its morphology studied over the multiscale curvature, geometry and texture (44). The image identification process carried out in this study included three components, which are image pre-processing, feature extraction and pattern recognition. The features were extracted automatically and used to compose a 3-dimensional feature vector for each oocyst image. Various problems were identified during this study, such as out of focus images, improperly positioned oocysts, compromised morphological structures, and the presence of debris and bacteria, which can make these variables difficult to use in the segmentation of the objects in question, impairing the identification of parasites (44).

*Eimeria* species are difficult to differentiate because of their highly similar morphology. However, a study using the Bayesian classifier was found to produce more accurate results than the SVM (Support Vector Machines). In fact, the Bayesian classifier presented 99.21% correct identification of *Eimeria maxima*, although *Eimeria necatrix* was incorrectly identified as *Eimeria acervulina* (6.10%) and as *Eimeria tenella* (9.94%). Moreover, 12.53% of *E. necatrix* was also mistaken for *E. acervulina*, 10.94% was mistaken for *Eimeria praecox*, and 12.22% for *E. tenella*. These results indicate that *E. necatrix* and *E. praecox* are the species most frequently identified, because their morphological similarity makes it difficult to differentiate them from each other and from other species. Nevertheless, in general, 85.75% of the parasites identified belonged to the genus *Eimeria* (44).

Researchers made a study based on Moment Invariants (MI) and a classifier of the Adaptive Neuro-Fuzzy Inference System (ANFIS) for the identification of 16 helminth parasite eggs in humans. This MI-ANFIS system consists of four stages: pre-processing, feature extraction, classification and testing. The ANFIS method, a more elaborate model of ANN, is considered a hybrid fuzzy logic algorithm and ANN. Therefore, the ANFIS classifier has all the advantages of these systems. The feature extraction stage uses Hu's seven moment invariants. The MI-ANFIS system showed a 93.49% correctness rate, and the main reason for incorrect classifications was the similarity in the shape of eggs, indicating the need for future advances in the technique (67).

The Multi-Class Support Vector Machine (MCSVM) classifier was tested in a study of 16 human parasites, which resulted in a 97.70% success rate. However, the same problem occurred as in the preceding study, i.e., the incorrect identification of similar shaped eggs. The pre-processing stage was considered the most important part of this proposal (68).

To improve the efficiency of existing conventional automated methods, a study was conducted using the MATLAB image processing toolbox (46). This study proposed a technique able to detect the presence of *Ascaris lumbricoides* and *Trichuris trichiura* parasites in a few seconds per image; however, this research was carried out only on these two parasite species (69).

In a routine laboratory parasitology diagnosis, debris, fecal impurities (41) and similarities in parasite structure (67, 68) pose real challenges for automated image analysis. Research has focused on the automatic segmentation and classification of microscopy images containing fecal impurities, and has detected the 15 most common species of protozoa, eggs and helminth larvae in Brazil. These species comprise *A. lumbricoides, Enterobius vermicularis, Ancylostomatidae, T. trichiura, Hymenolepis diminuta, Hymenolepis nana, Taenia* spp., *Schistosoma mansoni, Strongyloides stercoralis, Entamoeba histolytica* and *Entamoeba dispar, Giardia duodenalis, Entamoeba coli, Endolimax nana, Iodamoeba butschlii*, and *Blastocystis hominis*. Comparisons have been made of the performance of the OPF, ANN-MLP and SVM classifiers, with and without Bagging and AdaBoost, which are methods for building classifier committees. This evaluation demonstrated that the OPF classifier was the most suitable for the species in question, achieving 90.38% sensitivity, 98.32% specificity and 98.19% efficiency, with κ equal to 0.79 (41). For this study, a fecal sample processing technique was employed, called the TF-Test, which facilitates the concentration of parasite structures and help eliminate fecal impurities (41). In a later study, Suzuki et al. (41) proposed a complete solution for the diagnosis of intestinal parasites, with automated image acquisition from microscope slides and faster algorithms to reduce image processing time, and processed fecal samples using the TF-Test Modified technique (79). This study used an image base with 6,068 impurities and 1,791 parasites, and attained an average sensitivity of 93.00%, average specificity of 99.17% and average κ of 0.84 (80).

Based on a software program developed using morphometric analysis, area, perimeter and circularity, information on morphological specificity and characteristics of the parasites, 81.86% of the parasites were correctly identified. In that study, 85 images of *A. lumbricoides* and 54 images of *T. trichiura* were used. However, one of the main limitations of the automated technique is linked to debris and impurities from fecal samples left on microscope slides. This may explain the fact that the percentage of parasites not identified by automated means was 18.13%, which was attributed to the large amount of such impurities found in the evaluated samples (81).

Pattern classifiers are usually trained using a parasite image database annotated by a specialist. The automated reading of microscope slides can generate a large number of images to be annotated, rendering the process of manual annotation time-consuming and subject to errors. To facilitate this process, an active learning technique was developed whereby a specialist checks a small set of images, enabling the resulting classifier to automatically annotate the rest of the database. The proposed technique, called RDS (Root Distance-Based Sampling), organizes the dataset only once, as a pre-processing procedure, and adequately balances the diversity of classes as well as the sample uncertainty for the selection of useful samples during the learning process of a classifier, requiring verification of only a small part of the dataset (42).

A study of intestinal parasites was conducted using a cuvette-based automated microscopy analyzer, registered under the name of sediMAX 1®, which was developed for urine analysis. This equipment consists of a microscope, camera and high quality image processing software that can detect and classify particles in urine (82, 83). In this study, the equipment was used to automatically capture images from fecal samples, although the detection of parasites was performed by visual inspection. This device provides a practical way to store images for educational purposes, including the training of technicians in the detection of intestinal parasites (84).

In a study involving wastewater, a system was developed to identify and quantify up to seven species of helminth eggs. Images were captured manually using a microscope and color digital camera, and the system analyzed each image in <60 s. As in other studies of fecal samples, this study also came up against problems with debris and impurities that hindered the identification of eggs. Therefore, it is advisable to dilute concentrated sediment in tap water in a proportion of 1/1 or 1/2 (v/v). The system showed a detection specificity of 99% and its sensitivity varied from 80 to 90% (85).

The use of technology in the identification of intestinal parasites continues to expand rapidly and even involves mobile phones applications, as was the case with a study focusing on *A. lumbricoides*. In the study by Sowerby et al. (86), samples were processed by the flotation technique in saline solution and then identified using a mobile phone. Much still remains to be studied in the future, particularly the expansion of diagnostics for other parasites, the problem of impurities, and other challenges (86).

Using simple multivariate logistic regression, an algorithm was developed in the open-source program SCILAB to identify *Taenia* spp., *Fasciola hepatica, T. trichiura*, and *Diphyllobothrium latum* in stool smear images. The algorithm achieved sensitivity and specificity rates of 99.10 and 98.29% for *Taenia* spp., 99.15 and 98.18% for *Fasciola hepatica*, 100 and 98.38% for *T. trichiura*, and 100 and 98.13% for *D. latum*. A total of 200 samples were processed using rapid sedimentation (centrifugation) (87).

Aiming to provide automated treatment of gastrointestinal parasites in rural areas, a study was developed using a total of 30 microscope slides stained with iodine and containing eggs of *A. lumbricoides, T. trichiura* and hookworm species, in addition to four slides containing *Schistosoma haematobium*. These slides were digitized using a reference slide scanner and a mobile microscope. This new method aims to perform the diagnosis in less time but with high quality and accuracy, and at low cost. This proof-of-concept study demonstrates that the image of an inexpensive digital microscope suffices for a reliable diagnosis of the four helminth species that were worked on. Nevertheless, further studies are needed for improvement in order to overcome the challenges mentioned earlier herein (88).

Another analysis using the sediMAX® 2 was performed to compare the improvement achieved in the detection of protozoa in stool samples when compared with the sediMAX® 1. In this study, improvements were found in total reading time, which decreased from the original 10 min to about 5 min. SediMAX® 2 also allows amoebae to be differentiated by species, according to the number of nuclei present in the cells, as its focus is adjusted by hand. However, advances are still needed for an adequate diagnosis, such as the development of software for automatic image analysis, and to capture more than 15 images per sample (89).

Smartphones were also used in research to identify parasites, which are studied and analyzed for diagnostics in veterinary medicine. This research was based on *Strongylus* eggs found in horses, and an initial analysis indicated that the technique showed limitations in the identification of two eggs positioned close to each other or overlapping, and eggs covered by debris, making their identification difficult. This method was compared with the MiniFLOTAC and McMaster techniques, and was considered more sensitive than specific, generating false positive results; hence, further studies are still needed to improve this technique (90). A smartphone was also used in a study of three helminths that infect humans (*A. lumbricoides, T. trichiura* and hookworms), using Kankanet, an artificial neural network-based object detection smartphone application. The authors of the study reported sensitivity and specificity rates of 66.7 and 85.7% for *T. trichiura*, of 100 and 87.5% for *A. lumbricoides*, and of 100 and 100% for hookworms (91).

The use of automated diagnostics in the detection of intestinal parasites in various host species, such as sheep, canines, primates, and others was also developed for use in places where there are few resources. This is a portable method involving the McMaster flotation technique, which makes it low cost, fast and without requiring a trained professional to identify parasites. This technique attained good results, presenting an overall accuracy of 92 or 96% for *Eimeria* in the counting of one or four grids, respectively, and 100% for nematodes using one or four grids. In this study, debris was not a limitation, since the software was trained to recognize it (92).

Two classifiers were used in a study to identify eggs of the parasite *Ascaris* spp. in pigs, namely, the Multiclass Support Vector Machine (MC-SVM) and Artificial Neural Network (ANN). In this study, the parasite eggs were counted automatically. The accuracy rate of *Ascaris* spp. identification using the MC-SVM and ANN classifiers was ~95 and 93%, respectively (43).

In another study based on edge detection, image segmentation and recognition patterns, the detection and extraction of parasites in microscope images were fully automated, using the image pixel as a descriptor. This research made great strides in the detection of 15 human intestinal parasites, achieving an identification rate of 100% (93).

Staining also makes a significant difference in automated analysis for the more accurate identification of parasites. Thus, a convolutional neural network (CNN or ConvNet) model was developed to detect intestinal protozoa in human fecal samples stained with trichrome. However, this study showed limitations, such as the scantiness of some species. Even so, data from this study revealed that image capture using a slide scanner and Artificial Intelligence (AI) software allows for a 98.88% positive agreement [95% Confidence Interval from 93.76 to 99.98%], and 98.11% negative agreement [95% Confidence Interval from 93.35 to 99.77%] when compared to the correctness rates achieved with manual microscopy (94).

The performance of the VETSCAN IMAGYST system for the detection of parasites in dogs and cats was evaluated in another study, in which 100 fecal samples were analyzed, 84 from dogs and 16 from cats. The study revealed several limitations of the system, such as the lack of examination of the edges or outside the cover slip due to the reading area, and the study involved a low number of fecal samples, especially samples of *Trichuris* spp., *Toxocara* spp. and Taeniidae, which also limited the assessment of the system's diagnostic sensitivity and specificity (95).

In order to improve the diagnostic accuracy of the DAPI system (41) without compromising its efficiency and cost, a hybrid approach was proposed that combines two decision-making systems for the classification of images obtained from microscope slides. The study combines a simple system based on rapid extraction of characteristics from the images and SVM classifier, and a more complex system based on a deep neural network. The proposed system reached an average Kappa of 94.9, 87.8, and 92.5% in helminth eggs, helminth larvae and protozoan cysts, respectively (96).

A protocol designed to create a cleaner slide free of impurities contributed to a significant advance in the automated diagnosis of gastrointestinal parasites in the field of veterinary medicine. This technique was tested in a study involving four genera of canine intestinal parasites of high prevalence in an endemic region of the state of São Paulo, Brazil. Fecal samples from 104 dogs were collected to test this new protocol, which reached a Kappa index of 0.7636. It was therefore concluded that the new Prototic Coproparasitological Test for Dogs (PC-Test Dog) allows for a clearer view of parasite structures and presented a favorable result for the automated diagnosis of intestinal parasites in dogs (38).

More recently, Cringoli et al. (97) developed a portable versatile low-cost Kubic FLOTAC microscope (KFM) for students of veterinary medicine. The authors of the study prepared slides of bovine feces using the Mini-FLOTAC or FLOTAC method. Moreover, they stated that the KFM can be used to quantify parasite structures, and that the results were highly successful (97).

## Final Remarks

Fecal samples in which parasite structures are easily detectable make microscope slides easy to analyze (43). That is why most researchers use microscope images of fecal samples and use digital processing of technical images to eliminate fecal impurities and detect the presence of parasite structures (43). However, debris, impurities and parasite load are major limitations in the development of automated diagnostics.

Most of the studies described in this paper do not include an adequate protocol for the preparation of slides for use in automated diagnostics, which is a crucial aspect in the identification of intestinal parasites (96). To ensure the successful advance of automated parasitological diagnosis, a holistic view of the entire procedure must be adopted, from sample collection to identification on computers. In other words, samples must be collected and stored carefully, a processing technique should be used that reduces impurities and concentrates the parasites, as well as a suitable dye and a proper software program. These steps will undoubtedly be helpful in the advancements of automated diagnostics in both human and veterinary medicine.

## Author Contributions

SI, JG, and AF: conceptualization. SI: writing—original draft preparation. SI, BM, SR, CN, and KB: writing—review and editing. FS and SN: creation of the table. All the authors have read and agreed to the published version of the manuscript.

## Funding

This work was supported by Fundação de Amparo à Pesquisa do Estado de São Paulo (FAPESP) - Process No. 2017/14189-9 – Thematic Project Process No. 2014/12236-1 and National Council for Scientific and Technological Development (CNPQ) No. 303808/2018-7.

## Conflict of Interest

The authors declare that the research was conducted in the absence of any commercial or financial relationships that could be construed as a potential conflict of interest.

## Publisher's Note

All claims expressed in this article are solely those of the authors and do not necessarily represent those of their affiliated organizations, or those of the publisher, the editors and the reviewers. Any product that may be evaluated in this article, or claim that may be made by its manufacturer, is not guaranteed or endorsed by the publisher.
